# The complete chloroplast genome of *Apocynum venetum* (Apocynaceae)

**DOI:** 10.1080/23802359.2020.1781567

**Published:** 2020-06-26

**Authors:** Lianying Chang, Xiaolei Yu, Wenxiu Wang, Xiaoxuan Tian

**Affiliations:** a National Clinical Research Center for Chinese Medicine Acupuncture and Moxibustion, First Teaching Hospital of Tianjin University of Traditional Chinese Medicine, Tianjin, China; b Tianjin State Key Laboratory of Modern Chinese Medicine, Tianjin University of Traditional Chinese Medicine, Tianjin, China; c College of Pharmaceutical Engineering of Traditional Chinese Medicine, Tianjin University of Traditional Chinese Medicine, Tianjin, China

**Keywords:** *Apocynum venetum*, chloroplast genome

## Abstract

*Apocynum venetum* (*A. venetum*) has high medicinal value that belongs to the family Apocynaceae. Here, we reported the complete chloroplast (cp) genome of *A. venetum,* which was 150,858 bp in length. The cp genome was characterized by a typical quadripartite structure composed of a large single-copy region (LSC 81,919 bp) and a small single-copy region (SSC 17,257 bp) interspersed by a pair of 25,841 bp inverted repeat regions (IRs), and it contained 86 protein-coding genes, eight rRNAs, and 37 tRNAs. A maximum-likelihood (ML) phylogenetic tree indicated that *A. venetum* was closely related to *Trachelospermum jasminoides*.

*Apocynum venetum* is a wild plant of subshrub that belongs to the family Apocynaceae. It has a long history as a Chinese traditional medicine with uses to calm the liver, soothe the nerves, dissipate heat, and promote diuresis (Xie et al. 2012). Moreover, research on *A. venetum* showed that it has antidepressant potential and can reduce anxiety (Grundmann et al. 2007, 2009). To generate the genomic resources of this medicinal plant, we assembled and characterized the complete cp genome of *A. venetum*. This study will provide valuable resources for species identification, molecular biology, and phylogenetic studies of *A. venetum*.

Young fresh leaves of *A. venetum* were collected from Tianjin City (CHN, 38.96°N, 117.06°E). The voucher specimens were deposited in Tianjin State Key Laboratory of Modern Chinese Medicine, Tianjin University of Traditional Chinese Medicine, under the voucher number of LBM20200512. DNA isolation was carried out according to the standard protocol of Extract Genomic DNA Kit. Then, the libraries were sequenced with 2 × 150 bp on the Illumina HiSeq X Ten platform. The generated clean reads were used to assemble the cp genome of *A. venetum* using NOVOPlasty3.7.2 (Dierckxsens et al. 2017) with kmer length of 39. The cp genome of *A. venetum* was annotated using GeSeq (Tillich et al. 2017) coupled with manual correction for start and stop codons of protein-coding genes. Finally, the complete cp genome was submitted to GenBank under accession number MT547772.

The cp genome of *A. venetum* was 150,858 bp in length, including an LSC region of 81,919 bp, an SSC region of 17,257 bp, and a pair of IRs regions of 25,841 bp. Additionally, a total of 131 genes were annotated, including 86 protein-coding genes, eight rRNA genes, and 37 tRNA genes. Overall, the cp genome features of *A. venetum* were consistent with other angiosperms in terms of genomic structure (Cui et al. 2019; Yan et al. 2019; Yang et al. 2019; Yu et al. 2019).

To investigate the phylogenetic position of *A. venetum* in family Apocynaceae, *A. venetum* and other 16 Apocynaceae cp genome sequences available in GenBank were used to reconstruct the ML phylogenetic tree. The protein-coding genes were extracted, aligned separately, and recombined to construct a matrix using PhyloSuite_v1.1.15 (Zhang et al. 2020). Then, the matrix was used to conduct ML analyses. The ML tree was constructed using IQ-TREE (Nguyen et al. 2015) under the model automatically selected by IQ-TREE. The result indicated that *A. venetum* was closely related to *Trachelospermum jasminoides* (Figure 1).

**Figure 1.**
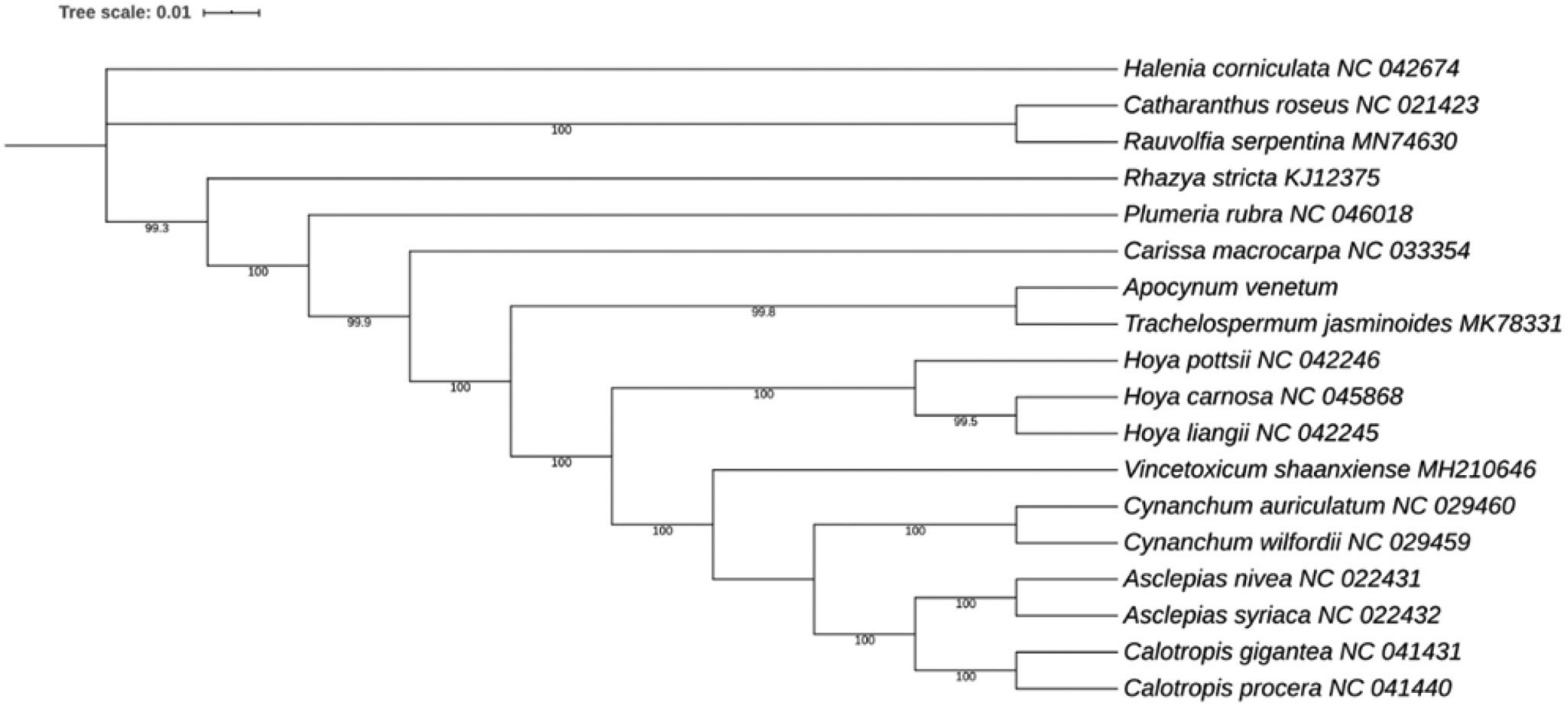
ML phylogenetic tree of Apocynaceae inferred from protein-coding genes of cp genomes. The *Halenia corniculata* was set as the outgroup. The numbers above the lines were bootstrap support values for ML analyses.

## Data Availability

The data that support the findings of this study is openly available in Genbank at https://www.ncbi.nlm.nih.gov/genbank/, reference number MT547772.
